# ﻿Molecular phylogeny and morphology reveal two new entomopathogenic species of *Ophiocordyceps* (Ophiocordycipitaceae, Hypocreales) parasitic on termites from China

**DOI:** 10.3897/mycokeys.103.116153

**Published:** 2024-03-08

**Authors:** Qi Fan, Tao Yang, Hui Li, Xue-Mei Wang, He-Fa Liao, Pei-Hong Shen, Zhu-Liang Yang, Wen-Bo Zeng, Yuan-Bing Wang

**Affiliations:** 1 College of Life Science and Technology, Guangxi University, Nanning 530004, Guangxi, China; 2 CAS Key Laboratory for Plant Diversity and Biogeography of East Asia, Kunming Institute of Botany, Chinese Academy of Sciences, Kunming 650201, Yunnan, China; 3 Yunnan Key Laboratory for Fungal Diversity and Green Development, Kunming Institute of Botany, Chinese Academy of Sciences, Kunming 650201, Yunnan, China; 4 College of Life Science, Yunnan University, Kunming 650091, Yunnan, China; 5 Notoginseng Medicine and Pharmacy, Wenshan University, Wenshan 663000, Yunnan, China

**Keywords:** New species, morphology, *Ophiocordyceps*, phylogeny, termites

## Abstract

Two new termite-pathogenic species, *Ophiocordycepsglobiperitheciata* and *O.longistipes*, are described from Yunnan Province, China. Six-locus (ITS, nrSSU, nrLSU, *tef-1α*, *rpb1* and *rpb2*) phylogenetic analyses in combination with morphological observations were employed to characterize these two species. Phylogenetically, *O.globiperitheciata* is most closely related to *Hirsutellacryptosclerotium* and *O.communis*, whereas *O.longistipes* shares a sister relationship with *O.fusiformis*. However, *O.globiperitheciata* differs from *H.cryptosclerotium* by parasitizing Blattodea and producing clavate, unbifurcated stromata. *Ophiocordycepsglobiperitheciata* is distinguished from *O.communis* by multiple stromata, shorter asci and ascospores. *Ophiocordycepslongistipes* differs from *O.fusiformis* in producing larger stromata, perithecia, asci and ascospores, as well as smaller citriform or oval conidia. Morphological descriptions of the two new species and a dichotomous key to the 19 termite-pathogenic *Ophiocordyceps* species are presented.

## ﻿Introduction

Invertebrate-associated fungi are intriguing and diverse, widely distributed around the world (Araújo et al. 2018; Luangsa-ard et al. 2018; Haelewaters and Kasson 2020; Wilson et al. 2021; Santamaria et al. 2023). There are two typical relationships between fungi and invertebrates. One is mutualism. Mutualism is reciprocally positive interactions between pairs of species (Bronstein 2009). For example, *Termitomyces* Heim (Lyophyllaceae, Agaricomycetes) can decompose plants to provide food for termites; in return, termites shelter *Termitomyces* from external threats (Da Costa et al. 2019). The other is parasitism. Parasitism is the interaction between two species where one party (the parasite) benefits, while the other party (the host) suffers harm (Roper et al. 2019). As exemplified by species of *Cordyceps* Fr. sensu lato (s. l.), fungi parasitize invertebrates and eventually kill them. Invertebrate-pathogenic fungi are considered as the most well-known parasitic fungi (Araújo et al. 2018; Araújo et al. 2021; Wilson et al. 2021). They are ubiquitous inhabitants of forests worldwide, especially in tropical and subtropical regions. Invertebrate-pathogenic fungi are highly virulent and are known to have significant effects on host populations (Evans 1974). *Cordyceps* s. l. represents the most abundant and diverse group among invertebrate-pathogenic fungi (Araújo et al. 2021). Representatives of this group can colonize hosts in more than 10 invertebrate orders (Sanjuan et al. 2015; Araújo and Hughes 2016). They spread primarily through their hosts, evolving extensively in their morphologies and parasitic strategies. (Araújo and Hughes 2016). According to the current status of *Cordyceps* s. l. taxonomy, it belongs to four families: Clavicipitaceae, Cordycipitaceae, Ophiocordycipitaceae and Polycephalomycetaceae (Sung et al. 2007a; Xiao et al. 2023). Among them, the genus *Ophiocordyceps* Petch (Ophiocordycipitaceae) has received significant attention for its unique interactions with hosts and medical values (Zou et al. 2017; Araújo et al. 2018; Luangsa-ard et al. 2018; Khonsanit et al. 2019; Wang et al. 2021a; Zou et al. 2022; Tang et al. 2023a).

*Ophiocordyceps* is the largest genus in the family Ophiocordycipitaceae (Araújo et al. 2018; Luangsa-ard et al. 2018). The genus was established by Petch based on the type species *O.blattae* Petch (Petch 1931). In recent years, an increasing number of species have been described in *Ophiocordyceps*, with approximately 410 accepted species names to date (http://www.indexfungorum.org/names/Names.asp) (Sung et al. 2007a; Sanjuan et al. 2015; Spatafora et al. 2015; Araújo et al. 2018; Evans et al. 2018; Luangsa-ard et al. 2018; Wijayawardene et al. 2018; Khonsanit et al. 2019; Mongkolsamrit et al. 2023; Tang et al. 2023a, b).

The majority of species in *Ophiocordyceps* exhibit clavate, entirely, or partially darkly pigmented stromata or synnemata, especially those species with a hirsutella-like anamorph, while some species possess brightly colored stromata with hymenostilbe-like anamorph. The stromata are mostly wiry, tough, leathery, and flexible. Perithecia are superficial to pseudo-immersed to fully immersed, and are vertically or obliquely inserted in the stromata. Asci are usually cylindrical with thickened apex and contain eight ascospores. Ascospores are typically cylindrical or clavate, multiseptate, either disarticulating into secondary spores or remaining whole after discharge (Sung et al. 2007a; Quandt et al. 2014; Luangsa-ard et al. 2018). Species in *Ophiocordyceps* mainly attack insects of Coleoptera, Diptera, Hemiptera, Hymenoptera, Lepidoptera, Odonata, and Orthoptera. Generally, they can attack all stages (larva, pupa, nymph, and adult) of the insects, with the majority of targets being larvae of Coleoptera and Lepidoptera living in wood or buried in soil (Sung et al. 2007a; Shrestha et al. 2016). Among species of *Ophiocordyceps*, only 17 species attack termites (Tasanathai et al. 2019; Araújo et al. 2021; Tang et al. 2022; Tasanathai et al. 2022; Xu et al. 2022).

Termites (Termitidae, Blattodea) are typically eusocial soil-dwelling insects, widely distributed around the world, especially in tropical and subtropical regions (Pearce 1997). Most termites are considered pests, causing significant impacts on forest ecosystems, and agricultural and forestry crops, with subterranean termites being particularly destructive (Rust and Su 2012; Scharf 2015). Some species of termite-pathogenic *Ophiocordyceps* have been regarded as potential biological agents to control termite populations (Rath 2000).

During surveys of invertebrate-pathogenic fungi in Yunnan Province, China, several specimens attacking termites were collected. Morphological and molecular evidence indicates that they belong to two different taxa distinct from previously described species. This study aims to introduce these two new species and discuss their evolutionary placement among related species.

## ﻿Materials and methods

### ﻿Collection and isolation

Stromata emerging above fallen leaves were found in subtropical evergreen broad-leaved forests of Ruili City and Jinghong City, Yunnan Province, China. Specimens were documented and photographed in the field using a Canon 90D digital camera, and then each was placed in a sterilized 50 mL plastic centrifugal tube. All samples were stored in a cooler with ice packs until they were taken to the laboratory. Pure cultures were obtained on potato dextrose agar (PDA) with the composition of 200 g/L potato, 20 g/L dextrose, and 20 g/L agar, following the method previously presented (Wang et al. 2020). Subsequently, pure cultures were transferred to PDA slants and stored at the Kunming Institute of Botany Culture Collection (**KUNCC**), Chinese Academy of Sciences. Dried specimens were deposited in the Cryptogamic Herbarium of the Kunming Institute of Botany, Chinese Academy of Sciences (**KUN-HKAS**).

### ﻿Morphological observations

The newly collected specimens were macroscopically examined with the Canon 750D camera and Olympus SZ60 stereo microscope. The characteristics of stromata (size, texture, shape, and color) were recorded. For the observation of teleomorph, perithecia were removed from the stromata and mounted on a glass slide with either 3% potassium hydroxide (KOH) (w/v) or 0.04% lactophenol cotton blue stain solution (w/v). Subsequently, the sizes and shapes of the perithecia, asci, and ascospores were measured under Olympus BX53 microscope. For each species, at least two specimens are measured, and each characteristic is measured at least 15 times repeatedly. The characteristics of pure cultures (size, texture, and color) were photographed using a Canon 750D camera after six weeks of culturing in an incubator at 25 °C. For the morphological description of anamorph, microscope slide cultures were prepared using the previous described method (Wang et al. 2020). Conidiogenous structures and conidia were measured and photographed using an Olympus BX53 microscope.

### ﻿DNA extraction, amplification and sequencing

Genomic DNA was extracted from fresh mycelia cultured for three weeks using Ezup Column Fungi Genomic DNA Extraction Kit (Sangon Bio Co., Ltd., Shanghai, China), following the manufacturer’s protocol. Polymerase chain reactions (PCRs) were used to amplify genetic markers using the following primer pairs: nrSSU-COF/nrSSU-COR for the nuclear ribosomal small subunits (nrSSU) (Wang et al. 2015), LR0R/LR5 for the nuclear ribosomal large subunits (nrLSU) (Vilgalys and Hester 1990; Hopple 1994), ITS5/ITS4 for the internal transcribed spacer (ITS) (White et al. 1990), EF1α-EF/EF1α-ER for the translation elongation factor 1α (*tef-1α*) (Bischoff et al. 2006; Sung et al. 2007b), RPB1-5F/RPB1-5R for the largest subunits of RNA polymerase II (*rpb1*) (Bischoff et al. 2006), and RPB2-5F/RPB2-7cR for the second largest subunits of RNA polymerase II (*rpb2*) (Bischoff et al. 2006; Sung et al. 2007b).

Each 25 µL-PCR reaction contained 12.5 µL of 2× Taq PCR Master Mix (Tiangen Biotech Co., Ltd., Beijing, China), 9.5 µL of RNase-Free water (Sangon Bio Co., Ltd., Shanghai, China), 1 µL of each forward and reverse primer (10 µmol/L), 1 µL of DNA template (500 ng/µL). PCR reactions were placed in a LongGene T20 multi-block thermal cycler (Hangzhou LongGene Scientific Instruments Co., Ltd., Hangzhou, China) under the following conditions: For ITS, (1) 3 min at 95 °C, (2) 36 cycles of denaturation at 94 °C for 30 sec, annealing at 55 °C for 50 sec and extension at 72 °C for 1 min, (3) extension at 72 °C for 5 min and 12 °C soak. For nrSSU, (1) 4 min at 95 °C, (2) 22 cycles of denaturation at 94 °C for 1 min, annealing at 51 °C for 1 min and extension at 72 °C for 90 sec, followed by (3) 12 cycles of denaturation at 94 °C for 1 min, annealing at 50 °C for 1 min and extension at 72 °C for 95 sec, (4) extension at 72 °C for 10 min and 12 °C soak. For nrLSU, (1) 4 min at 95 °C, (2) 36 cycles of denaturation at 94 °C for 1 min, annealing at 50 °C for 1 min and extension at 72 °C for 2 min, (3) extension at 72 °C for 10 min and 12 °C soak. For *tef-1α*, (1) 3 min at 95 °C, (2) 36 cycles of denaturation at 94 °C for 30 sec, annealing at 50 °C for 30 sec and extension at 72 °C for 1 min, (3) extension at 72 °C for 10 min and 12 °C soak. For *rpb1*, (1) 4 min at 95 °C, (2) 36 cycles of denaturation at 94 °C for 40 sec, annealing at 50 °C for 40 sec and extension at 72 °C for 90 sec, (3) extension at 72 °C for 10 min and 12 °C soak. For *rpb2*, (1) 3 min at 95 °C, (2) 36 cycles of denaturation at 94 °C for 45 s, annealing at 58 °C for 45 s and extension at 72 °C for 90 s, (3) extension at 72 °C for 10 min and 12 °C soak. Standard DNA markers (Sangon Bio Co., Ltd., Shanghai, China) of known size and weight were used to quantify the PCR products. PCR products were purified using the DiaSpin PCR Product Purification Kit (Sangon Bio Co., Ltd., Shanghai, China), following the manufacturer’s instructions. Purified PCR products were sent to Sangon Bio Co., Ltd., (Kunming, China) for Sanger sequencing. The newly generated sequences were checked using MEGA v. 7.0 (Kumar et al. 2016). Consensus sequences were obtained using SeqMan of the Lasergene software package v. 14.1 (DNAstar, Madison, Wisconsin, USA) and deposited in NCBI GenBank (https://www.ncbi.nlm.nih.gov/genbank).

### ﻿Sequencing alignments and phylogenetic analyses

We generated sequences for six loci from five specimens (Table 1). These were complemented with sequences of 125 related samples downloaded from NCBI GenBank based on BLAST searches and recent publications on Ophiocordycipitaceae (Tang et al. 2022; Xu et al. 2022). *Tolypocladiuminflatum* Gams OSC 71235 and *T.ophioglossoides* (J.F. Gmel.) Quandt et al. CBS 100239 were selected as the outgroup. The sequence datasets were aligned using MAFFT v. 7, and alignments were manually corrected in MEGA v. 7.0 (Katoh and Standley 2013; Kumar et al. 2016). Ambiguously aligned sites were manually eliminated and gaps were regarded as missing data. ModelFinder (Kalyaanamoorthy et al. 2017) was used to select the best-fit nucleotide substitution models for Maximum Likelihood (ML) and Bayesian Inference (BI) analyses under the Akaike Information Criterion (AIC). The optimized models for each locus partition are presented in Table 2. Partitioned ML and BI analyses were performed on the concatenated data set. The BI analysis was conducted using the MrBayes v. 3.2 (Ronquist et al. 2012). Four simultaneous Markov chains were run for 2,000,000 generations with a sub-sampling frequency every 100 generations. A burn-in of the first 25% of the total run was discarded. ML analysis was conducted using IQ-TREE v. 2.1.3 (Nguyen et al. 2015) under partitioned models (Chernomor et al. 2016) with 1000 ultrafast bootstrap (Hoang et al. 2018). Trees were visualized with its Maximum-Likelihood bootstrap proportions (ML-BS) and Bayesian posterior probability (BI-PP) in FigTree v. 1.4.4 and edited with Adobe Illustrator CS6.0.

**Table 1.** Voucher information and GenBank accession numbers for the sequences included in this study.

Species	Voucher information	GenBank accession no.						Reference
		ITS	nrSSU	nrLSU	*tef-1α*	*rpb1*	*rpb2*	
*Hirsutellasatumaensis*	ARSEF 996	–	KM652082	KM652125	KM652008	KM652047	–	Simmons et al. 2015
H.cf.haptospora	ARSEF 2228	KM652166	KM652075	KM652118	KM652001	KM652041	–	Simmons et al. 2015
*H.citriformis*	ARSEF 1446	KM652154	KM652065	KM652106	KM651990	KM652031	–	Simmons et al. 2015
*H.cryptosclerotium*	ARSEF 4517	KM652157	KM652066	KM652109	KM651992	KM652032	–	Simmons et al. 2015
*H.fusiformis*	ARSEF 5474	–	KM652067	KM652110	KM651993	KM652033	–	Simmons et al. 2015
*H.gigantea*	ARSEF 30	–	–	JX566977	JX566980	KM652034	–	Simmons et al. 2015
*H.guyana*	ARSEF 878	–	KM652068	KM652111	KM651994	KM652035	–	Simmons et al. 2015
*H.haptospora*	ARSEF 2226	KM652159	–	–	KM651995	KM652036	–	Simmons et al. 2015
*H.illustris*	ARSEF 5539	KM652160	KM652069	KM652112	KM651996	KM652037	–	Simmons et al. 2015
*H.kirchneri*	ARSEF 5551	–	KM652070	KM652113	KM651997	–	–	Simmons et al. 2015
*H.lecaniicola*	ARSEF 8888	KM652162	KM652071	KM652114	KM651998	KM652038	–	Simmons et al. 2015
*H.liboensis*	ARSEF 9603	KM652163	KM652072	KM652115	–	–	–	Simmons et al. 2015
*H.necatrix*	ARSEF 5549	KM652164	KM652073	KM652116	KM651999	KM652039	–	Simmons et al. 2015
*H.nodulosa*	ARSEF 5473	KM652165	KM652074	KM652117	KM652000	KM652040	–	Simmons et al. 2015
*H.radiata*	ARSEF 1369	–	KM652076	KM652119	KM652002	KM652042	–	Simmons et al. 2015
*H.repens* nom. inval.	ARSEF 2348	KM652167	KM652077	KM652120	KM652003	–	–	Simmons et al. 2015
*H.rhossiliensis*	ARSEF 3747	KM652168	KM652080	KM652123	KM652006	KM652045	–	Simmons et al. 2015
*H.strigosa*	ARSEF 2197	KM652174	KM652085	KM652129	KM652012	KM652050	–	Simmons et al. 2015
*H.subulata*	ARSEF 2227	KM652176	KM652086	KM652130	KM652013	KM652051	–	Simmons et al. 2015
*H.thompsonii*	ARSEF 257	KM652182	–	KM652139	KM652019	KM652056	–	Simmons et al. 2015
	ARSEF 414	KM652184	–	KM652143	KM652021	KM652059	–	Simmons et al. 2015
H.thompsoniivar.vina	ARSEF 254	–	KM652101	KM652149	KM652028	KM652062	–	Simmons et al. 2015
*H.versicolor*	ARSEF 1037	–	KM652102	KM652150	KM652029	KM652063	–	Simmons et al. 2015
*Ophiocordycepsacicularis*	OSC 110988	–	EF468951	EF468804	EF468745	EF468853	–	Sung et al. 2007a
*O.agriotidis*	ARSEF 5692	JN049819	DQ522540	DQ518754	DQ522322	DQ522368	DQ522418	Spatafora et al. 2007
*O.annulata*	CEM 303	–	KJ878915	KJ878881	KJ878962	KJ878995	–	Quandt et al. 2014
*O.appendiculata*	NBRC 106960	JN943326	JN941728	JN941413	AB968577	JN992462	AB968539	Schoch et al. 2012
*O.arborescens*	NBRC 105891	AB968398	AB968386	AB968414	AB968572	–	AB968534	Ban et al. 2015
*O.asiana*	MY11878	MW285719	–	MW280213	MW292448	MW296049	–	Khao-ngam et al. 2021
*O.asiatica*	BCC 30516	MH754722	–	MH753675	MK284263	MK214105	MK214091	Tasanathai et al. 2019
	BCC 86435	MH754723	–	MH753676	–	MK214106	MK214092	Tasanathai et al. 2019
*O.barnesii*	BCC 28560	–	EU408776	–	–	EU408773	EU418599	Luangsa-ard et al. 2010
*O.bidoupensis*	YFCC 8793	–	OM304638	–	OK556894	OK556898	OK556900	Zou et al. 2022
*O.brunneinigra*	BCC 69032	–	–	MF614654	MF614638	MF614668	MF614681	Luangsa-ard et al. 2018
*O.brunneiperitheciata*	BCC 66167	–	–	MF614659	MF614644	–	MF614684	Luangsa-ard et al. 2018
*O.brunneipunctata*	OSC 128576	–	DQ522542	DQ518756	DQ522324	DQ522369	DQ522420	Spatafora et al. 2007
*O.brunneirubra*	BCC 14384	MH754736	–	MH753690	GU797121	MK751465	MK751468	Tasanathai et al. 2019
*O.campes*	BCC 36938	MT783955	–	MT118175	MT118167	MT118183	MT118188	Tasanathai et al. 2020
*O.communis*	BCC 1842	MH754726	–	MH753680	MK284266	MK214110	MK214096	Tasanathai et al. 2019
	BCC 1874	MH754725	–	MH753679	MK284267	MK214109	MK214095	Tasanathai et al. 2019
	BCC 2754	MH754727	–	MH753681	MK284268	MK214111	MK214097	Tasanathai et al. 2019
*O.cossidarum*	MFLU 17-0752	–	MF398186	MF398187	MF928403	MF928404	–	Xiao et al. 2019
*O.crinalis*	GDGM 17327	–	KF226253	KF226254	KF226256	KF226255	–	Wang et al. 2014
*O.dipterigena*	OSC 151911	–	KJ878919	KJ878886	KJ878966	KJ879000	–	Quandt et al. 2014
*O.elongata*	OSC 110989	–	–	EF468808	EF468748	EF468856	–	Sung et al. 2007a
*O.flavida*	BCC 84256	–	–	MT512655	MT533482	MT533476	–	Mongkolsamrit et al. 2021
*O.formosana*	TNM F13893	–	KJ878908	–	KJ878956	KJ878988	KJ878943	Quandt et al. 2014
*O.furcatosubulata*	YFCC 902	–	MT774214	MT774221	MT774242	MT774228	MT774235	Wang et al. 2021b
*O.fusiformis*	BCC 93025	MZ676743	–	MZ675422	MZ707849	MZ707855	MZ707805	Tasanathai et al. 2022
	BCC 93026	MZ676744	–	MZ675423	MZ707850	MZ707856	MZ707806	Tasanathai et al. 2022
*O.geometridicola*	BCC 35947	–	–	MF614647	MF614631	MF614664	MF614678	Luangsa-ard et al. 2018
*O.globiceps*	MFLU 18-0661	MH725816	MH725812	MH725830	MH727388	–	–	Xiao et al. 2019
***O.globiperitheciata***	**HKAS 126130**	**OR015963**	**OR082950**	**OR015968**	**OR030532**	**OR119834**	–	**This study**
	**HKAS 126131**	**OR015964**	**OR082951**	**OR015969**	**OR030533**	**OR119835**	–	**This study**
*O.globosa*	BCC 93023	MZ676740		MZ675419	MZ707846	MZ707861	–	Tasanathai et al. 2022
*O.halabalaensis*	MY5151	GU723763	KM655826	–	GU797110	–	–	Luangsa-ard et al. 2011
*O.hydrangea*	YFCC 8832	–	OM304636	OM304640	OM831277	OM831280	OM831283	Zou et al. 2022
*O.irangiensis*	BCC 82795	MH028142	–	–	MH028186	MH028164	MH028174	Khonsanit et al. 2019
*O.isopterae*	MY12376	MZ676741	–	MZ675420	MZ707847	MZ707859	MZ707803	Tasanathai et al. 2022
	BCC 93042	MZ676742	–	MZ675421	MZ707848		MZ707804	Tasanathai et al. 2022
*O.karstii*	MFLU 15-3884	–	KU854952	–	KU854945	KU854943	–	Li et al. 2016
*O.khokpasiensis*	BCC 48071	MH754728	–	MH753682	MK284269	MK214112	–	Tasanathai et al. 2019
	BCC 48072	MH754729	–	MH753683	MK284270	MK214113	–	Tasanathai et al. 2019
	BCC 1764	MH754730	––	MH753684	MK284271	MK214114	MK214098	Tasanathai et al. 2019
	BCC 81464	MK632043	MK632128	MK632103	MK632077	MK632170	MK632159	Tasanathai et al. 2019
*O.kimflemingiae*	SC09B	–	KX713631	KX713620	KX713698	KX713724	–	Araújo et al. 2018
*O.konnoana*	EFCC 7315	–	EF468959	–	EF468753	EF468861	EF468916	Sung et al. 2007a
*O.longissima*	EFCC 6814	–	–	EF468817	EF468757	EF468865	–	Sung et al. 2007a
	NBRC 106965	AB968406	AB968392	AB968420	AB968584	–	AB968546	Ban et al. 2015
***O.longistipes***	**KUNCC 5224**	**OR015962**	**OR082949**	**OR015967**	**OR030530**	**OR062224**	**OR113082**	**This study**
	**HKAS 126186**	**OR015960**	**OR082947**	**OR015966**	**OR030531**	**OR062225**		**This study**
	**HKAS 126187**	**OR015961**	**OR082948**	**OR015965**	**OR030529**	**OR062223**		**This study**
*O.longistromata*	BCC 44497	MT783956	–	MT118178	MT118170	–	MT118191	Tasanathai et al. 2020
*O.macroacicularis*	NBRC 100685	AB968400	AB968388	AB968416	AB968574	–	AB968536	Ban et al. 2015
*O.megacuculla*	BCC 82984	–	–	MH028162	MH028192	–	MH028181	Khonsanit et al. 2019
*O.mosingtoensis*	BCC 30904	MH754732	–	MH753686	MK284273	MK214115	MK214100	Tasanathai et al. 2019
*O.mosingtoensis*	BCC 36921	MH754731	–	MH753685	MK284272	MK214116	MK214099	Tasanathai et al. 2019
*O.multiperitheciata*	BCC 22861	–	–	MF614656	MF614640	MF614670	MF614683	Luangsa-ard et al. 2018
*O.myrmecophila*	CEM 1710	–	–	KJ878894	KJ878974	KJ879008	–	Quandt et al. 2014
*O.nigrella*	EFCC 9247	JN049853	EF468963	EF468818	EF468758	EF468866	EF468920	Sung et al. 2007a
*O.nutans*	OSC 110994	–	DQ522549	DQ518763	DQ522333	DQ522378	–	Spatafora et al. 2007
*O.ovatospora*	YHH 2206001	OP295105	OP295110	OP295113	OP313801	OP313803	OP313805	Tang et al. 2022
	YFCC 22069184	OP295106	OP295111	OP295114	OP313802	OP313804	–	Tang et al. 2022
*O.pauciovoperitheciata*	TBRC 8096	–	–	MF614649	MF614636	MF614665	MF614672	Luangsa-ard et al. 2018
*O.phuwiangensis*	BCC 85351	MT783958	–	–	MT118174	MT118187	MT118195	Tasanathai et al. 2020
	BCC 86208	–	–	MT118180	MT118172	MT118185	MT118193	Tasanathai et al. 2020
*O.pruinosa*	NHJ 12994	–	EU369106	EU369041	EU369024	EU369063	EU369084	Johnson et al. 2009
*O.pseudoacicularis*	BCC 53843	–	–	MF614646	MF614630	MF614661	MF614677	Luangsa-ard et al. 2018
*O.pseudocommunis*	NHJ 12581	–	EF468973	EF468831	EF468775	–	EF468930	Luangsa-ard et al. 2018
	NHJ 12582		EF468975	EF468830	EF468771	–	EF468926	Luangsa-ard et al. 2018
*O.pseudocommunis*	BCC 16757	MH754733	–	MH753687	MK284274	MK214117	MK214101	Tasanathai et al. 2019
*O.pseudolloydii*	MFLUCC 15-0689	MF351725	–	–	MF372758	MF372761	–	Xiao et al. 2017
*O.pseudorhizoidea*	BCC 48879	MH754720	–	MH753673	MK284261	MK214104	MK214089	Tasanathai et al. 2019
	BCC 86431	MH754721	–	MH753674	MK284262	MK751469	MK214090	Tasanathai et al. 2019
	NHJ 12522	JN049857	–	EF468825	EF468764	EF468873	EF468923	Tasanathai et al. 2019
	NHJ 12529	–	–	EF468824	EF468765	EF468872	EF468922	Tasanathai et al. 2019
*O.puluongensis*	YFCC 6442	–	MT141118	MT270528	MT270520	MT270523	MT270526	Xu et al. 2022
	YFCC 6443	–	MT141119	MT270529	MT270521	MT270524	MT270527	Xu et al. 2022
	YHH 16017	–	–	MT270530	MT270522	MT270525	–	Xu et al. 2022
*O.pulvinata*	TNS-F 30044	AB721302	GU904208	–	GU904209	GU904210	–	Kepler et al. 2011
*O.radiciformis*	BCC 93036	MZ676746	–	MZ675425	MZ707852	MZ707857	MZ707808	Tasanathai et al. 2022
	BCC 93035	MZ676747	–	MZ675426	MZ707853	MZ707858	MZ707809	Tasanathai et al. 2022
*O.ramosissimum*	GZUHHN8	KJ028007	KJ028012	–	KJ028014	KJ028017	–	Wen et al. 2014
*O.ravenelii*	OSC 110995	–	DQ522550	DQ518764	DQ522334	DQ522379	DQ522430	Spatafora et al. 2007
*O.rhizoidea*	NHJ 12522	JN049857	EF468970	EF468825	EF468764	EF468873	EF468923	Tasanathai et al. 2019
	NHJ 12529	–	EF468969	EF468824	EF468765	EF468872	EF468922	Tasanathai et al. 2019
*O.robertsii*	KEW 27083	AJ309335	–	EF468826	EF468766	–	–	Sung et al. 2007a
*O.rubiginosiperitheciata*	NBRC 106966	JN943344	JN941704	JN941437	AB968582	JN992438	AB968544	Kepler et al. 2012
*O.salganeicola*	Mori01	–	MT741705	MT741719	MT759575	MT759578	MT759580	Araújo et al. 2021
	Mori02	–	MT741704	MT741718	MT759572	MT759579	MT759581	Araújo et al. 2021
*O.satoi*	J7	–	KX713653	KX713599	KX713683	KX713711	–	Araújo et al. 2018
*O.sinensis*	ARSEF 6282	KM652173	KM652083	KM652126	KM652009	KM652048	–	Simmons et al. 2015
	EFCC 7287	JN049854	EF468971	EF468827	EF468767	EF468874	EF468924	Kepler et al. 2012
*O.sobolifera*	NBRC 106967	AB968409	AB968395	AB968422	AB968590	–	–	Ban et al. 2015
*O.spataforae*	NHJ 12525	–	EF469125	EF469078	EF469063	EF469092	EF469111	Sung et al. 2007a
	OSC 128575	JN049845	EF469126	EF469079	EF469064	EF469093	EF469110	Sung et al. 2007a
*O.sphecocephala*	NBRC 101752	JN943351	JN941696	JN941445	AB968591	JN992430	AB968552	Schoch et al. 2012
*O.spicatus*	MFLU 18-0164	MK863254	MK863047	MK863054	MK860192	–	–	Zha et al. 2021
*O.stylophora*	OSC 111000	JN049828	DQ522552	DQ518766	DQ522337	DQ522382	DQ522433	Spatafora et al. 2007
	OSC 110999	–	EF468982	EF468837	–	EF468882	EF468931	Sung et al. 2007a
*O.termiticola*	BCC 1920	MH754724	–	MH753678	MK284265	MK214108	MK214094	Tasanathai et al. 2019
	BCC 1770	GU723780	–	MH753677	MK284264	MK214107	MK214093	Tasanathai et al. 2019
	BCC 93002	–	–	MZ675427	MZ707854	MZ707862	MZ707810	Tasanathai et al. 2019
*O.thanathonensis*	MFLU 16-2909	MF850376	–	MF850377	MF872613	MF872615	–	Xiao et al. 2017
*O.tricentri*	NBRC 106968	AB968410	AB968393	AB968423	AB968593	–	AB968554	Ban et al. 2015
*O.unilateralis*	OSC 128574	–	DQ522554	DQ518768	DQ522339	DQ522385	DQ522436	Spatafora et al. 2007
*O.unituberculata*	YFCC HU1301	–	KY923214	–	KY923216	KY923218	KY923220	Wang et al. 2018
*O.xuefengensis*	GZUHHN13	KC631804	KC631785	–	KC631790	KC631795	–	Wen et al. 2013
	GZUH2012HN13	KC631801	KC631787	–	KC631792	KC631797	–	Wen et al. 2013
*trichospora*	CBS 109876	–	AF543766	AF543790	AF543779	AY489669	DQ522457	Sung et al. 2007a
*Tolypocladiuminflatum*	OSC 71235	JN049844	EF469124	EF469077	EF469061	EF469090	EF469108	Sung et al. 2007b
*T.ophioglossoides*	CBS 100239	–	KJ878910	KJ878874	KJ878958	KJ878990	KJ878944	Quandt et al. 2014

Note: Newly-generated sequences are shown in bold.

**Table 2.** Results of the best-ftting model for maximum likelihood (ML) and Bayesian inference (BI) for six loci partitions.

Gene name	ML	BI
ITS	GTR+F+I+G4	GTR+F+I+G4
nrSSU	TNe+I+G4	SYM+I+G4
nrLSU	TIM+F+I+G4	GTR+F+I+G4
*tef-1α*	TIM2+F+I+G4	GTR+F+I+G4
*rpb1*	TIM+F+I+G4	GTR+F+I+G4
*rpb2*	TIM3+F+I+G4	GTR+F+I+G4

## ﻿Results

### ﻿Phylogenetic analyses

**Figure 1.**
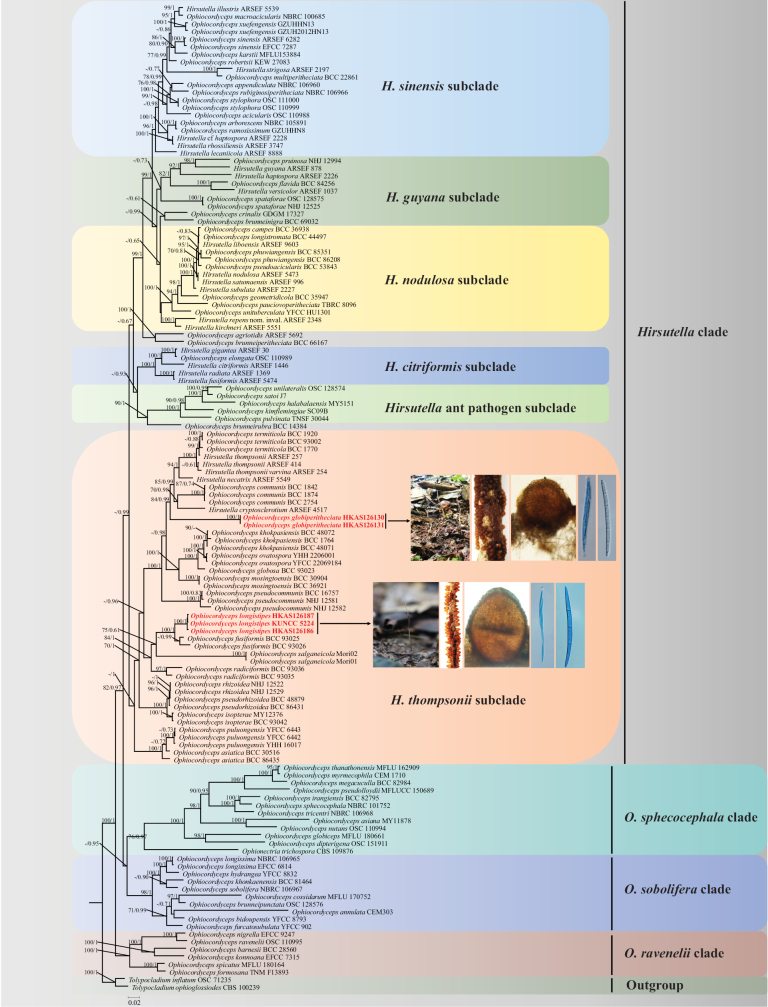
Phylogenetic tree based on the combined dataset of nrSSU, nrLSU, *tef-1α*, *rpb1*, *rpb2*, and ITS showing the relationship of two new species on termites from China with other *Ophiocordyceps* species. Values at the nodes before and after the backslash are BI posterior probabilities (BI-PP greater than 0.60) and ML bootstrap proportions (ML-BP greater than 70%), respectively. New species described in this paper are shown in bold red.

The combined dataset of six loci was composed of 5021 bp (585 bp for ITS, 903 bp for nrLSU, 1037 bp for nrSSU, 859 bp for *tef-1α*, 664 bp for *rpb1*, and 973 bp for *rpb2*). Phylogenetic trees inferred from ML and BI analyses exhibited nearly consistent overall topologies and recognized four statistically well-supported clades within *Ophiocordyceps*, namely *Hirsutella* Pat, *O.sphecocephala* (Klotzsch ex Berk.) Sung et al., *O.sobolifera* (Hill ex Watson) Sung et al., and *O.ravenelii* (Berk. & M.A. Curtis) Sung et al. clades (Fig. 1). Among them, the *Hirsutella* clade includes six distinct subclades, namely *H.citriformis* Speare, *H.thompsonii* Fisher, *H.nodulosa* Petch, *H.guyana* Minter & Brady, *H.sinensis* (Berk.) Sung et al., and the *Hirsutella* ant pathogen subclades. As revealed from phylogenetic analyses, all specimens collected in this study were placed in the *H.thompsonii* subclade. Three samples (HKAS 126185, HKAS 126186, and HKAS 126187), newly described as *O.longistipes*, were clustered closely with *O.fusiformis* Tasan et al. However, the phylogenetic evidence indicated that these three samples formed a monophyletic clade in *Ophiocordyceps*, with high statistical support (ML-BS/BI-PP=100/1). The other two samples (HKAS 126130 and HKAS 126131), newly described as *O.globiperitheciata*, clustered together and formed a separate clade, distinguishing from other species in *Ophiocordyceps* with moderate bootstrap support (ML-BS/BI-PP=84/0.99). Therefore, the phylogenetic data supported the recognition of *O.longistipes* and *O.globiperitheciata* as distinct species in *Ophiocordyceps*.

### ﻿Taxonomy

#### ﻿ Ophiocordyceps longistipes

Taxon classificationFungiHypocrealesOphiocordycipitaceae

Y.B. Wang, T. Yang, Q. Fan & Zhu L. Yang sp. nov.

CBABB789-0E59-5460-8EFB-97C7F203A012

Index Fungorum: IF901029

Fig. 2

##### Etymology.

Referring to the long stipe of stromata.

##### Type.

***Holotype***: China, Yunnan Province, Ruili City, 26°1.07'N, 97°51.33'E, alt. 1140 m, on a termite buried in soil, 2 July 2022, Tao Yang (holotype HKAS 126185, ex-type culture KUNCC 5224). Ex-type sequences (ITS: OR015962, nrLSU: OR015967, nrSSU: OR082949, *tef-1α*: OR030530, *rpb1*: OR062224, *rpb2*: OR113082).

##### Description.

Stromata arising from the back of termites buried in soil, solitary, unbranched, cylindrical, flexible, leathery, 17–24 cm long, 0.5–1.0 mm wide, grayish white to yellowish brown. Fertile parts cylindrical, yellowish brown, 3–5.5 cm long, generating toward the upper part of stromata, covered by a spinous surface, with a sterile tip of 11–28 × 0.5–1.0 mm. Perithecia superficial, pale yellow at early stage, brown at maturity, pyramidal to oval, densely distributed in the upper of stromata, arranged in a disordered manner, 390–420 × 295–350 µm. Asci 8-spored, filiform, hyaline, 160–195 × 4.5–6.5 µm, with hemispheric apical cap. Ascospores whole, hyaline, filiform, tapering at both ends, 70–85 × 3.5–4.5 µm, multiseptate, septa 4.5–13.8 μm long.

##### Anamorph.

hirsutella-like. Colonies on PDA growing very slowly, reaching 3–3.5 cm diam after six weeks at 25 °C, felty, irregularly convex, cream, reverse pale brown to dark brown. Hyphae hyaline, branched, septate, smooth-walled, 2–3 µm wide. Conidiogenous cells arising from aerial mycelia, monophialidic or rarely polyphialidic, on hyphae laterally or terminally, hyaline, smooth, flask-shaped, 29–60 μm long, with a swollen base, 4–4.5 μm wide, tapering sharply into a thin neck, 0.5–0.8 μm wide. Conidia borne directly on the tip of phialides, hyaline, one-celled, solitary, smooth-walled, citriform or oval, 7–10 × 4.5–7 µm, with a mucous sheath.

**Figure 2.**
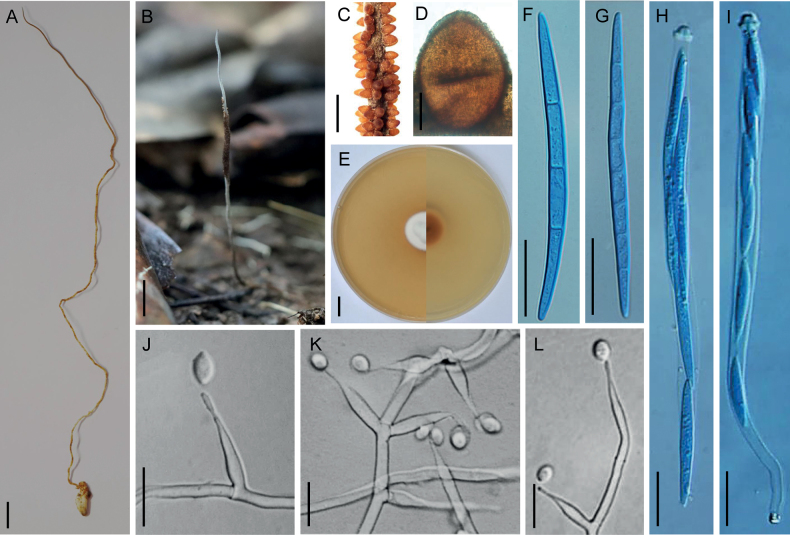
*Ophiocordycepslongistipes***A, B** stromata of fungus arising from termites **C** fertile part **D** perithecia **E** colony on PDA (obverse and reverse) **F, G** ascospores **H, I** asci **J–L** conidiogenous cells and conidia. Scale bars: 1 cm (**A, B, E**); 2 mm (**C**); 100 µm (**D**); 20 µm (**F–L**).

##### Additional specimens examined.

China, Yunnan Province, Ruili City, 26°1.07'N, 97°51.33'E, alt. 1140 m, on a termite buried in soil, 2 July 2022, Tao Yang (HKAS 126186), sequences (ITS: OR015960, nrLSU: OR015966, nrSSU: OR082947, *tef-1α*: OR030531, *rpb1*: OR062225). *Ibid.*, (HKAS 126187), sequences (ITS: OR015961, nrLSU: OR015965, nrSSU: OR082948, *tef-1α*: OR030529, *rpb1*: OR062223).

##### Habitat and ecology.

Parasitic on termites buried in soil of the subtropical evergreen broad-leaved forests, emerging from fallen leaves on the forest floor.

##### Known distribution.

Ruili City, Yunnan Province, China.

##### Notes.

*Ophiocordycepslongistipes* is characterized by solitary stromata, superficial and pyramidal to oval perithecia, filiform asci, and filiform ascospores, hirsutella-like anamorph with monophialidic or rarely polyphialidic, flask-shaped conidiogenous cells, and citriform or oval conidia embedded in a mucous sheath. Phylogenetically, all specimens of *O.longistipes* are clustered in the *H.thompsonii* subclade of *Hirsutella* lineages and form a monophyletic clade, which is placed sister to *O.fusiformis* with maximum support (Fig. 1). However, *O.longistipes* exhibits significant morphological differences from *O.fusiformis* in its both teleomorph and anamorph. For the teleomorph, *O.longistipes* produce longer stromata of 17–24 cm (up to 6 cm long for *O.fusiformis*), larger perithecia of 390–420 × 295–350 µm (300–360 × 180–270 µm for *O.fusiformis*). For the anamorph, *O.longistipes* possess both monophialidic and polyphialidic conidiogenous cells, but *O.fusiformis* is only monophialidic. Moreover, *O.longistipes* produces oval conidia, while *O.fusiformis* produces narrower fusiform conidia (Table 3).

**Table 3.** Morphological comparison between *Ophiocordyceps* species parasitic on termites.

Species	Host	Stromata (cm)	Perithecia (µm)	Asci (µm)	Ascospore (µm)	Conidiogenous cells (µm)	Conidia (µm)	Reference
*O.asiatica*	Termites	Solitary, simple, filiform, orange brown, up to 15 long	Superficial, globose to subglobose, 240–320 × 180–260	Filiform, 92.5–175 × 5–6.3	Filiform, septate, whole, 80–132.5 × 1–2	Monophialidic or rarely polyphialidic, 15–20 × 2–3	Fusiform, 7–9 × 2–3	(Tasanathai et al. 2019)
*O.bispora*	Termites	Multiple (20–30), simple or branched, clavate	Immersed, globose, 300–375 × 375	Clavate, 162–163 × 58–61	Elliptical closely appressed, septate, 95–105 × 34–35.4			(Blackwell and Gilbertson 1984; Ochiel et al. 1997)
*O.brunneirubra*	Termites	Solitary, simple or branched, narrowly clavate, orange brown to red brown, 9.5 long	Immersed, ovoid, 300–400 × 130–200	Cylindrical, 155–225 × 4.5–8	Filiform, septate, whole, 156.5–197.5 × 2–3	Monophialidic, 32–50 × 2–3	Fusiform, 12–17 × 2–4	(Tasanathai et al. 2019)
*O.communis*	Termites	Solitary, simple, filiform, base whitish-grey, upper part yellow-brown, 5–13 long	Superficial, 285–675 × 195–390	Filiform, 215–250 × 15	Filiform, septate, whole, 100–180 × 5–6	Monophialidic or rarely polyphialidic, 10–14 × 2.7–3.3	Almond-shaped, 7–9 × 2.5–3	(Sung et al. 2007a)
*O.fusiformis*	Termite	Solitary, simple, cylindrical, brown, up to 6 long	Superficial, ovoid, 300–360 × 180–270	Cylindrical, 141–227 × 7–15	Cylindrical, septate, whole, 36–78 × 5–6.5	Monophialidic, 9–24 × 2–4	Fusiform, 6–18 × 2–4	(Tasanathai et al. 2022)
*O.globosa*	Termites	Solitary, simple, cylindrical, brown, up to 8 long	Pseudo-immersed, ovoid, 190–245 × 120–190	Filiform, 100–157 × 7–13	Filiform, septate, whole, 58–118 × 2–3	Monophialidic or polyphialidic, 9–15 × 3–5	Globose, 2–4	(Tasanathai et al. 2022)
***O.globiperitheciata***	**Termites**	**Multiple (2–5), unbifurcated, clavate, base brown, tip gray, 8–15 long**	**Superficial, subglobose, 240–295 × 215–280**	**Filiform, 135–170 × 8.5–13.5**	**Filiform, septate, whole, 85–110 × 3.5–4.5**			**This study**
*O.isopterae*	Termites	Solitary, simple, cylindrical, brown, up to 10 long	Superficial, ovoid, 270–320 × 140–180	Filiform, 81–137 × 5–9	Filiform, septate, whole, 55–78 × 2–2.5	Monophialidic, 14–28 × 2–4	Fusiform, 6–11 × 1.5–3	(Tasanathai et al. 2022)
*O.khokpasiensis*	Termites	Solitary, simple, cylindrical, brown, 16 long	Pseudo-immersed, subglobose, 200–250 × 120–200	Filiform, 62.5–125 × 4–5	Filiform, whole, 46–90 × 2–3	Monophialidic or polyphialidic, 15–28 × 3–5	Globose to oval, 4–6 × 2.5–4	(Khonsanit et al. 2019)
*O.koningsbergeri*	Termites	Solitary, filiform, gray-white, 8–10 long	Immersed, 450 × 90	Cylindrica, 180–200 × 4–5	Filiform, whole, 150 × 1			(Penzig and Saccardo 1904)
***O.longistipes***	**Termites**	**Solitary, unbifurcated, cylindrical, grayish white to yellowish brown, 17–24 long**	**Superficial, pyramidal to oval, 390–420 × 295–350**	**Filiform, 160–195 × 4.5–6.5**	**Filiform, septate, whole, 70–85 × 3.5–4.5**	**Monophialidic or rarely polyphialidic, on hyphae laterally or terminally, 29–60 long, with a swollen base, 4–4.5 wide, tapering sharply into a thin neck, 0.5–0.8 wide.**	**Citriform or oval, 7–10 × 4.5–7**	**This study**
*O.mosingtoensis*	Termites	Solitary, simple, cylindrical, brown to grey, 11 long	Pseudo-immersed, ovoid, 400–500 × 200–300	Filiform, 187.5–287.5 × 4.5–7.5	Filiform, septate, whole, 230–315 × 1.5–3	Monophialidic, 10–17 × 2–3	Oval, 3–5 × 2–3	(Tasanathai et al. 2019)
*O.octospora*	Termites	Multiple, clavate, white to pale tan, 0.2–0.3 long	Immersed, subglobose to ovoid, 180–220 × 200	Clavate, about 250 × 60	Cylindrical, septate, 40–70 × 15–30			(Blackwell and Gilbertson 1981)
*O.ovatospora*	Termites	Solitary, simple, cylindrical or clavate, light-yellow, up to 13 long	Pseudo-immersed, ovoid to pyriform, 110–140 × 80–110	Filiform, 110–125 × 5–7	Filiform, septate, whole, 110–130 × 1–2	Monophialidic or rarely polyphialidic, 15–35 × 3–6	Oval, 3–5 × 3–4	(Tang et al. 2022)
*O.pseudocommunis*	Termites	Solitary, simple, cylindrical, brown, 21 long	Superficial, Subglobose, 520–600 × 360–440	Filiform, 160–165 × 14–17	Filiform,septate, whole, 107.5–147.5 × 6–7.5	Arising from hyphae laterally or terminally	Fusiform, septate (2–3), 13–27 × 3–5	(Tasanathai et al. 2019)
*O.pseudorhizoidea*	Termites	Solitary, simple, filiform, light brown, up to 21 long	Superficial, ovoid, 280–390 × 160–220	Cylindrical, 120–150 × 5–7	Filiform, whole, 65–82.5 × 2–3	Monophialidic, 9–21 × 2–4	Fusiform, 5–10 × 1–2	(Tasanathai et al. 2019)
*O.puluongensis*	Termites	Solitary, simple or branched, filiform, pale orange to red brown, 7.1–13.3 long	Superficial, subglobose, 181.8–251.0 × 123.7–205.4	Fliform, 74.3–138.5 × 4.6–6.5	Filiform, septate, whole, 67.0–124.5 × 1.5–2.5	Monophialidic or rarely polyphialidic, 7.9–21.2 × 1.7–5.0	Fusiform or citriform, 2.8–6.1 × 1.9–3.4	(Xu et al. 2022)
*O.radiciformis*	Termites	Solitary, simple, cylindrical, brown, up to 11 long	Superficial, ovoid, 330–460 × 200–320	Cylindrical, 140–296 × 6–10	Filiform septate, whole, 154–215 × 2–3	6–15 × 2–5	Fusiform, 5–7 × 2–3	(Tasanathai et al. 2022)
*O.termiticola*	Termites	Solitary, simple, filiform, yellow brown, up to 14 long	Pseudo-immersed, globose to subglobose, 200–280 × 150–250	Filiform 62.5–110 × 4–6	Filiform, whole, 85 × 2	Monophialidic to polyphialidic, 7–11 × 2.5–4	Globose, 2.5–3.5	(Tasanathai et al. 2019)

#### ﻿ Ophiocordyceps globiperitheciata

Taxon classificationFungiHypocrealesOphiocordycipitaceae

Y.B. Wang, T. Yang, Q. Fan & Zhu L. Yang sp. nov.

825A1CAC-16CC-5671-BD8C-95405C4B9186

Index Fungorum: IF901030

Fig. 3

##### Etymology.

Referring to the shape of perithecia, with “*globi*” meaning globose.

##### Type.

***Holotype***: China, Yunnan Province, Jinghong City, Puwen Town, 22°26.35'N, 101°1.32'E, alt. 970 m, on a termite buried in soil, 28 Sep. 2022, Tao Yang (HKAS 126130). Holotype sequences (ITS: OR015963, nrLSU: OR015968, nrSSU: OR082950, *tef-1α*: OR030532, *rpb1*: OR119834).

##### Description.

Stromata arising from the termite buried in soil, multiple (2–5), clavate, unbranched, flexible, leathery, 8–15 cm long, 1–1.5 mm wide, tapering from base to tip, base brown, tip gray. Fertile parts cylindrical, pale brown, generating toward the upper part of stromata, covered by a spinous surface, with a sterile tip. Perithecia superficial, pale brown to brown, subglobose, aggregating loosely at the upper of stromata, arranged in a disordered manner, 240–295 × 215–280 µm. Asci 8-spored, filiform, hyaline, 135–170 × 8.5–13.5 µm, with a hemispheric apical cap. Ascospores whole, hyaline, tapering at both ends, filiform, 85–110 × 3.5–4.5 µm, multiseptate, septa 11–14.5 μm long. Anamorph not detected.

**Figure 3.**
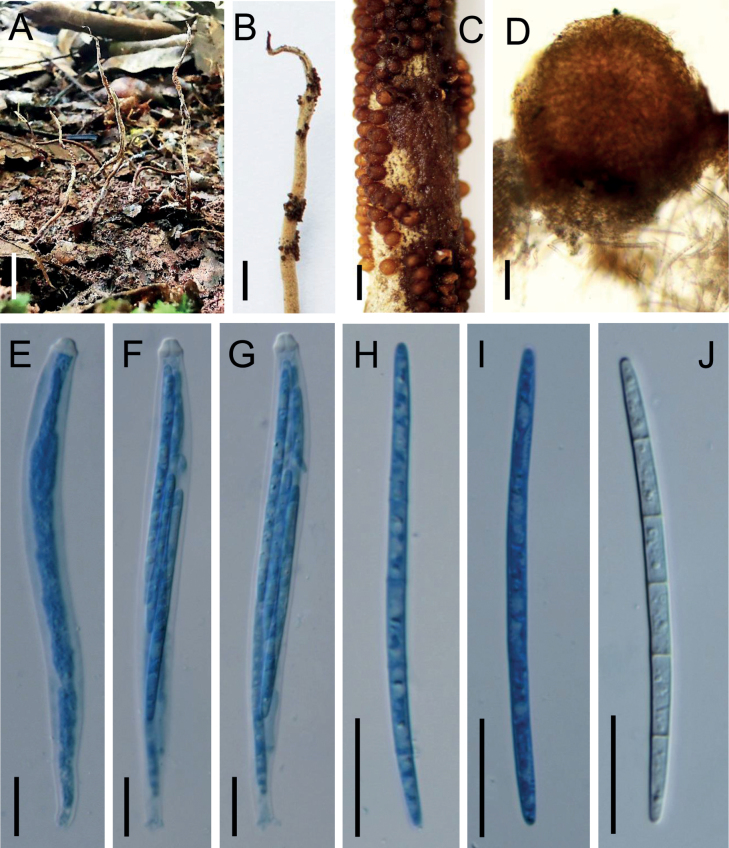
*Ophiocordycepsglobiperitheciata***A** stromata of fungus arising from termites **B** sterile tip and fertile part **C** fertile part **D** perithecia **E–G** asci **H–J** ascospores. Scale bars: 1 cm (**A**); 2 mm (**B**); 500 µm (**C**); 50 µm (**D**); 20 µm (**E–J**).

##### Additional specimens examined.

China, Yunnan Province, Jinghong City, Puwen Town, 22°26.35'N, 101°1.32'E, alt. 970 m, on a termite buried in soil, 28 Sep. 2022, Tao Yang (HKAS 126131). Sequences (ITS: OR015964, nrLSU: OR015969, nrSSU: OR082951, *tef-1α*: OR030533, *rpb1*: OR119835).

##### Habitat and ecology.

Parasitic on termites buried in soil of tropical evergreen broad-leaved forests, emerging from fallen leaves on the forest floor.

##### Known distribution.

Puwen Town, Jinghong City, Yunnan Province, China.

##### Notes.

*Ophiocordycepsglobiperitheciata* is characterized by multiple and unbranched stromata, superficial and subglobose perithecia, and filiform asci and ascospores. Phylogenetically, *O.globiperitheciata* forms a separate clade from other *Ophiocordyceps* species in the *H.thompsonii* subclade with moderate bootstrap support (Fig. 1). It is closed to *H.cryptosclerotium* Fern. et al. and *O.communis* Hywel-Jones & Samson. However, it differs from *H.cryptosclerotium* in parasitizing Blattodea (*H.cryptosclerotium* parasitic on Hemiptera), producing multiple clavate stromata (*H.cryptosclerotium* stroma absence). *Ophiocordycepsglobiperitheciata* is distinguished from *O.communis* by multiple and thicker stromata, shorter asci of 135–170 µm (215–250 µm for *O.communis*) and ascospores of 85–110 µm (100–180 µm for *O.communis*) (Table 3).

### ﻿Key to species of *Ophiocordyceps* parasitic on termites

**Table:** 

1	Stromata multiple	**2**
–	Stromata solitary	**4**
2	Perithecia superficial	***O.globiperitheciata***
–	Perithecia immersed	**3**
3	Perithecia subglobose to ovoid	***O.octospora***
–	Perithecia globose	***O.bispora***
4	Perithecia nonsuperficial	**5**
–	Perithecia superficial	**11**
5	Perithecia immersed	**6**
–	Perithecia pseudo-immersed	**7**
6	Stromata orange brown to red brown	***O.brunneirubra***
–	Stromata gray-white	***O.koningsbergeri***
7	Only monophialidic	***O.mosingtoensis***
–	Possessing polyphialidic	**8**
8	Large asci (100–160 µm long)	***O.globosa***
–	Small asci (60–130 µm long)	**9**
9	Large ascospores (> 100 µm long)	***O.ovatospora***
–	Small ascospores (< 100 µm long)	**10**
10	Conidia globose	***O.termiticola***
–	Conidia globose to oval	***O.khokpasiensis***
11	Stromata sometimes branched	***O.puluongensis***
–	Stromata unbranched	**12**
12	Long stromata (≥ 15 cm long)	**13**
–	Short stromata (< 15 cm long)	**16**
13	Conidia have septa	***O.pseudocommunis***
–	Conidia have no septa	**14**
14	Short stromata (< 16 cm long)	***O.asiatica***
–	Long stromata (> 16 cm long)	**15**
15	Long conidiogenous cells (> 25 µm long)	***O.longistipes***
–	Short conidiogenous cells (< 25 µm long)	***O.pseudorhizoidea***
16	Conidia almond-shaped	***O.communis***
–	Conidia fusiform	**17**
17	Short asci (< 140 µm long)	***O.isopterae***
–	Long asci (≥ 140 µm long)	**18**
18	Long stromata (> 6 cm long)	***O.radiciformis***
–	Short stromata (≤6 cm long)	***O.fusiformis***

## ﻿Discussion

Thus far, only 17 species of *Ophiocordyceps* parasitic on termites were described, mainly clustered in the *H.thompsonii* subclade (Tasanathai et al. 2019; Tasanathai et al. 2022). These species are: *O.asiatica* Tasanathai et al., *O.bispora* (Stifler) G.H. Sung et al., *O.brunneirubra* Tasanathai et al., *O.communis* Hywel-Jones & Samson, *O.fusiformis* Tasanathai et al., *O.globosa* Tasanathai et al., *O.isopterae* Tasanathai et al., *O.khokpasiensis* Tasanathai et al., *O.koningsbergeri* (Penz. & Sacc.) G.H. Sung et al., *O.mosingtoensis* Tasanathai et al., *O.octospora* (M. Blackw. & Gilb.) G.H. Sung et al., *O.ovatospora* H. Yu et al., *O.pseudocommunis* Tasanathai et al., *O.pseudorhizoidea* Tasanathai et al., *O.puluongensis* H. Yu et al., *O.radiciformis* Tasanathai et al., and *O.termiticola* Tasanathai et al. Most the termite-pathogenic *Ophiocordyceps* species are found in tropical and subtropical regions, which may be related to the higher diversity of both *Ophiocordyceps* fungi and their termite hosts in these climatic zones (Sung et al. 2007a; Tasanathai et al. 2019; Cerezer et al. 2020; Araújo et al. 2021; Wilson et al. 2021; Tang et al. 2022; Tasanathai et al. 2022; Xu et al. 2022).

Phylogenetically, almost all *Ophiocordyceps* species parasitic on termites are placed in the *H.thompsonii* subclade, except for *O.brunneirubra*. Termite-pathogenic species exhibit significant morphological variation overall. Among these species, the length of stromata ranges from extremely short to very long, the existence pattern of perithecia from superficial to pseudo-immersed to immersed, and the size of perithecia ranges from about 100 to 600 µm (Tasanathai et al. 2019; Araújo et al. 2021; Tasanathai et al. 2022; Xu et al. 2022). However, some of these species exhibit minimal interspecific morphological variation, making it challenging to distinguish them only through morphological studies. Therefore, the use of molecular systematics is necessary to accurately identify these species. For example, *O.asiatica* and *O.puluongensis*, as well as *O.khokpasiensis* and *O.termiticola*, share similar morphological characteristics. *Ophiocordycepsasiatica* and *O.puluongensis* produce subglobose superficial perithecia, similar asci, ascospores, conidiogenous cells, and conidia (Tasanathai et al. 2019; Xu et al. 2022). *Ophiocordycepskhokpasiensis* and *O.termiticola* possess similar colored and shaped stromata, pseudo-immersed perithecia, and similar asci, ascospores, and conidiogenous cells (Tasanathai et al. 2019). Although these species are morphologically indistinguishable, phylogenetic analyses support them as separate taxa.

It’s worth noting that the hosts of these termite-pathogenic *Ophiocordyceps* species are usually buried underground, typically 5 to 15 cm below the ground, which may be relevant to the subterranean living habits of the host termites (Martelossi et al. 2023). However, this can pose a challenge for species identification, as hosts are often lost due to separation from fragile stromata during excavation (Tasanathai et al. 2022).

Termites are notorious pests known for damaging wood, cultivated plants, buildings, pastures, forests, and even non-cellulosic materials like cables, causing annual economic losses amounting to tens of billions of dollars. Subterranean termites are responsible for about 80% of the total damage (Rust and Su 2012; Scharf 2015; Oi 2022). Therefore, the control of termites has become the focus of attention in various industries. Previously, many chlorinated hydrocarbon insecticides were used for termite control, but they were banned due to their irreversible environmental impact and negative effects on crop production. Consequently, environmentally friendly and sustainable control measures for termites are urgently needed. Entomopathogenic fungi may represent a potent solution (Afzal et al. 2019; Tasanathai et al. 2019; Oi 2022; Moon et al. 2023). These fungi, with strong infectivity, can continuously spread spores in the field to control pests and are considered environmentally non-polluting, so they have significant advantages in pest control (Shimazu et al. 1995; Meyling and Eilenberg 2007). Most members of *H.thompsonii* subclade have been found to obligately parasitize termites, they may have a regulatory effect on natural termite populations. Particularly, *O.bispora*, for which field investigations have revealed a high infection rate against termites, and laboratory experiments have also shown that it can effectively kill termites (Blackwell and Gilbertson 1984; Suh et al. 1998). Although laboratory experiments have not been conducted with *O.longistipes*, field observations have found that termites infected by this fungus often appear in groups. This may indicate that it has strong lethality against termites and possesses the potential to become a biological control agent for termites.

## Supplementary Material

XML Treatment for Ophiocordyceps longistipes

XML Treatment for Ophiocordyceps globiperitheciata

## ﻿Additional information

### Author ORCIDs

Qi Fan https://orcid.org/0000-0003-3168-0347

Tao Yang https://orcid.org/0009-0000-8579-1574

Hui Li https://orcid.org/0000-0003-4255-8312

Xue-Mei Wang https://orcid.org/0009-0001-0171-4924

He-Fa Liao https://orcid.org/0009-0006-7287-1384

Pei-Hong Shen https://orcid.org/0000-0003-0980-9562

Zhu-Liang Yang https://orcid.org/0000-0001-9745-8453

Wen-Bo Zeng https://orcid.org/0009-0000-0251-5890

Yuan-Bing Wang https://orcid.org/0000-0002-3305-9418

### Data availability

All of the data that support the findings of this study are available in the main text.
